# Fluorescent Sensing of Glutathione and Related Bio-Applications

**DOI:** 10.3390/bios13010016

**Published:** 2022-12-23

**Authors:** Xiaohuan Sun, Fei Guo, Qianyun Ye, Jinfeng Zhou, Jie Han, Rong Guo

**Affiliations:** School of Chemistry and Chemical Engineering, Yangzhou University, Yangzhou 225002, China

**Keywords:** glutathione, fluorescent sensing, cancer diagnosis, bio-applications

## Abstract

Glutathione (GSH), as the most abundant low-molecular-weight biological thiol, plays significant roles in vivo. Abnormal GSH levels have been demonstrated to be related to the dysfunction of specific physiological activities and certain kinds of diseases. Therefore, the sensing of GSH is emerging as a critical issue. Cancer, with typical high morbidity and mortality, remains one of the most serious diseases to threaten public health. As it is clear that much more concentrated GSH is present at tumor sites than at normal sites, the in vivo sensing of GSH offers an option for the early diagnosis of cancer. Moreover, by monitoring the amounts of GSH in specific microenvironments, effective diagnosis of ROS levels, neurological diseases, or even stroke has been developed as well. In this review, we focus on the fluorescent methodologies for GSH detection, since they can be conveniently applied in living systems. First, the fluorescent sensing methods are introduced. Then, the principles for fluorescent sensing of GSH are discussed. In addition, the GSH-sensing-related biological applications are reviewed. Finally, the future opportunities in in the areas of fluorescent GSH sensing—in particular, fluorescent GSH-sensing-prompted disease diagnosis—are addressed.

## 1. Introduction

Glutathione (GSH) plays crucial roles in physiological processes, such as protecting cells from oxidative damage, maintaining intracellular redox homeostasis, signal transduction, etc. [1,2,3,4]. Abnormal levels of GSH have been demonstrated to be related to the dysfunction of a series of biological activities, as well as certain kinds of diseases—for example, Alzheimer’s disease or cancer [5,6]. Taking the above facts into account, the accurate sensing of GSH—especially in vivo—is of significant importance [7,8,9]. To date, a number of methods have been developed for the detection of GSH, including mass spectroscopy [10], high-performance liquid chromatography [11,12], nuclear magnetic resonance [13], colorimetric methods [14,15], electrochemical methods [16,17,18], enzymatic methods [19], and fluorescent approaches [20,21]. Among all of these methods, fluorescent methodologies have attracted significant attention due to their abundant output signals, high sensitivity, and great potential for in vivo imaging [22]. 

Cancer, as one of the most malignant diseases, is the second-leading cause of death globally. According to the World Health Organization, delayed cancer treatment decreases the chance of survival, induces cancer metastases and, of course, leads to a great deal of physical, emotional, and financial burdens on patients [23,24]. Therefore, the early diagnosis of cancer remains an urgent issue. In addition to screening, the most common strategy for cancer diagnosis is the sensing of characteristic markers. Considering that the GSH concentration has been demonstrated to be much higher in the microenvironment of cancer (2–10 mM) than that of normal sites (2–20 μM), the approach of GSH sensing—especially when taking advantage of the fluorescent method—is rationally further encouraged for cancer diagnosis [7,9]. The above idea can be applied to other GSH-related diseases as well. 

**Figure 1:**
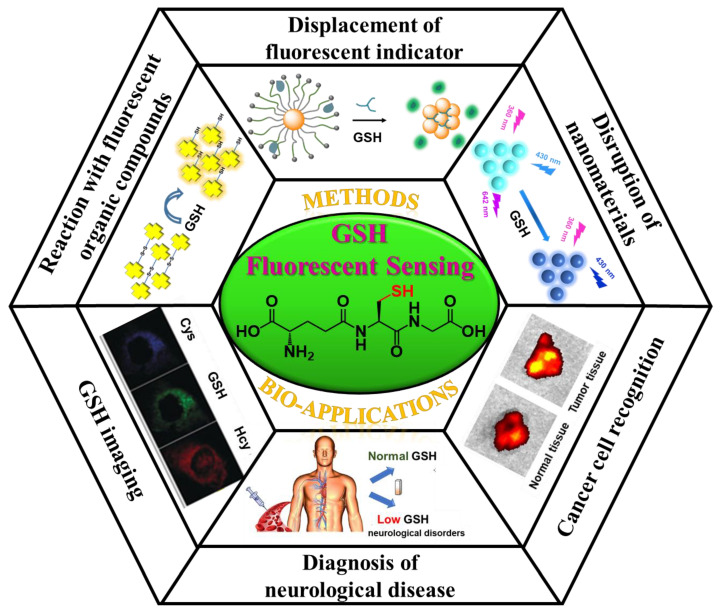
Schematic representation of the fluorescent GSH sensing methods and related bio-applications.

Taken together, in this review, the underlying mechanisms for fluorescent sensing are first introduced. Afterwards, the principles for the design of fluorescent GSH sensors according to the various origins of fluorescent output signals are discussed (Figure 1) [25]. Then, the recent progress of fluorescent GSH-sensing-related bio-applications (such as imaging, cancer diagnosis, etc.) is exemplified [26]. Finally, the future prospects for fluorescent GSH sensing and corresponding bio-applications are described. It is believed that the review presented herein will clarify the design principle for fluorescent GSH sensors and, more importantly, open up new horizons for GSH-sensing-actuated bio-applications. 

## 2. Fluorescent Sensing Principle

### 2.1. The Components of Fluorescent Sensors

The development of chemosensors has been an essential topic among scientists, as it significantly benefits the areas of public safety, clinical diagnosis, environmental protection, etc. [27,28]. It is well known that common chemosensors are composed of a recognition element, transduction element, and signal processing element. By determining the output signals, the chemosensors can be categorized as electrochemical sensors [29], colorimetric sensors [30], NMR sensors [31,32,33], fluorescent chemosensors [34,35,36], etc. Among them, fluorescent sensors have attracted plenty of attention recently [37]. In this section, the design principle for fluorescent sensors is briefly introduced, and representative mechanisms are exemplified. 

The recognition unit is one of the most important factors that needs to be considered for the fabrication of fluorescent sensors [38]. The most basic prerequisite for the design of a recognition unit is that it should have the ability to interact with analytes. Though this requirement is arbitrary, the interaction mode between the recognition unit and analytes can be flexible, e.g., covalent reaction [39], electrostatic interaction [40,41], hydrogen bonding interaction [42], Van der Waals interaction [43,44], hydrophobic interaction [45,46], π–π stacking [47,48], etc. Taking advantage of the above methods, the analytes can be selectively reacted, absorbed, or bound with the fluorescent sensor from a bulk phase. After the capture of the analytes, the interacting event between the recognition unit and the analyte—which can result in changes in the molecular/nanomaterial structure or the initiation/disruption of specific self-assembly behavior—is transferred to the signal processing element (a fluorophore, in the case of fluorescent sensors) through the transduction element, which normally serves as the link between the recognition element and the fluorophore [49]. In general, the fluorescent signal alteration induced by the above stimuli can be divided into on–off, off–on, on–off–on, and off–on–off modes. Nevertheless, the nature of the fluorescent intensity change lies in the alteration of the processing of electronic excited states of the fluorophore, which can be manipulated by photoinduced electron transfer (PET) [50], intramolecular charge transfer (ICT) [51], fluorescence resonance energy transfer (FRET) [52], excited-state intramolecular proton transfer (ESIPT), etc. 

### 2.2. The Mechanisms of Fluorescent Sensors

**Figure 2:**
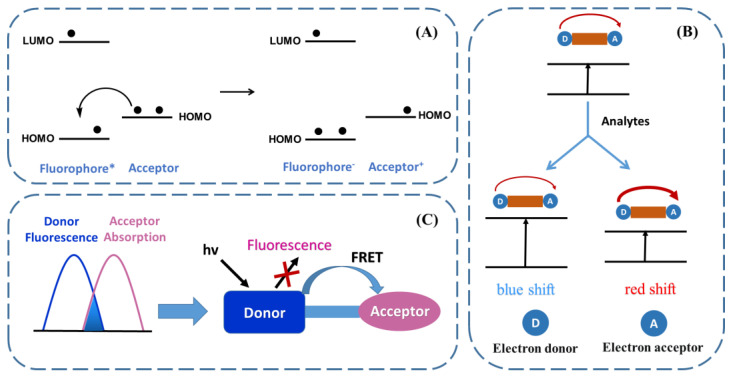
Schematic representation of the (**A**) PET, (**B**) ICT, and (**C**) FRET processes (reproduced with permission from [53], Copyright 2020, Elsevier B.V.).

The PET process is a common strategy for the development of off–on fluorescent sensors (Figure 2). Essentially, for the related probe, the fluorophore is connected with a recognition acceptor, usually through the transduction unit. Before its interaction with the analytes, the highest occupied molecular orbital (HOMO) of the acceptor is higher than that of the fluorophore. When irradiated, an electron from the HOMO of the fluorophore is excited to the lowest unoccupied molecular orbital (LOMO), promoting the charge transfer from the HOMO of the acceptor to the HOMO of the fluorophore and quenching the fluorescence, providing a fluorescence-off initial state. However, upon the interaction with the analytes, the energy level of the HOMO of the acceptor will be lower than that of the fluorophore, preventing the PET process and leading to the emission of fluorescence [53]. 

The ICT process occurs in the system where the fluorophore is simultaneously linked with an electron-donating group and an electron-withdrawing group (Figure 2B) [51]. With photoirradiation, the ICT takes place and results in different dipole moments, along with the characteristic fluorescence emission originating from the ICT state. Upon the interaction with either the electron-donating group or the electron-withdrawing group, the presence of analytes can modulate the fluorescence spectrum through the alteration of the original dipole moment. Specifically, after interacting with the analytes, either the electron-donating capability of the donating group or the electron-withdrawing capability of the withdrawing group is improved, and the fluorescent spectrum will be redshifted. Otherwise, a blueshifted fluorescence spectrum will be obtained. 

The FRET process is defined as the dipole–dipole interaction of the overlapped orbitals between the ground state of an acceptor and the excited state of a donor (Figure 2C) [54]. There are several requirements for the occurrence of the FRET, including the proximity of the donor and acceptor and the overlap between the excited spectrum of the acceptor and the emission spectrum of the donor [55]. Once the FRET efficiency is sensitive to the binding between the fluorescent probe and the analytes, the change in the fluorescence intensity or emission spectrum of the probe will take place. According to the FRET, on–off–on-type fluorescence sensors known as indicator displacement assays have been developed [56]. In these cases, highly emissive fluorescent compounds or nanomaterials are chosen as indicators. Through the regulation of the interaction of the indicators with the FRET acceptor, the fluorescence of the indicators can be quenched. When in the presence of the analytes, which exhibit higher affinity for the FRET acceptor, the indicators were displaced by analytes from the surface of the FRET acceptor, leaving the fluorescence turned on [57,58]. 

## 3. Fluorescent Methods for GSH Sensing

GSH, the tripeptide of γ-L-glutamyl-L-cysteinyl-glycine, is well known for its vital biological functions [59]. Due to its ability to interact with fluorescent species, compounds, or nanomaterials, GSH can be quantitatively evaluated via the changing of fluorescent signals. According to the origin of the output signals, the recent progress in fluorescent GSH sensing can be divided into three categories: (1) The output signals originate from fluorescent compounds. Through chemical reaction with fluorescent compounds, GSH can induce changes in the fluorescence intensity or fluorescence emission wavelength. (2) The output signals originate from fluorescent nanomaterials. Through the disruption of the surface properties or nanostructure of the nanomaterials, GSH can lead to the alteration of the fluorescence performance. (3) The output signals originate from fluorescent indicators. Through the interruption of the FRET between the fluorescent indicators and certain sensing nanoplatforms, GSH can switch on the fluorescence of indicators. In this section, the working principles and corresponding mechanisms for each kind of GSH fluorescent sensor are discussed. Additionally, the pros and cons of these methodologies are clarified. 

### 3.1. Fluorescent GSH Sensing via Its Reaction with Organic Fluorescent Compounds 

The organic fluorescent compounds are common species that can be employed for fluorescent GSH sensing [60,61]. The related mechanism lies in the reaction between GSH—usually the sulfhydryl group with strong nucleophilic and reductive characteristics—and the recognition unit of the organic fluorescent compounds, such as nucleophilic substitution reaction, nucleophilic addition reaction, reduction reaction, etc. [62,63,64]. 

**Figure 3:**
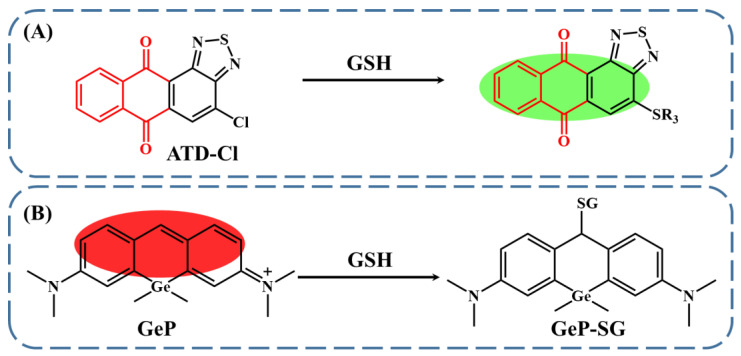
The fluorescent GSH sensing with (**A**) ATD-Cl (reproduced with permission from [65], Copyright 2018, Elsevier B.V.) and (**B**) GeP, taking advantage of the nucleophilic characteristics of GSH (reproduced with permission from [66], Copyright 2022, Elsevier B.V.).

From the above perspective, the 4-chloro-anthra [1,2-c] [1,2,5] thiadiazole-6,11–dione (ATD-Cl) was synthesized by Liu et al. (Figure 3A) [65]. They deliberately introduced an electron-withdrawing group of -Cl on the skeleton of the fluorophore to prevent the ICT process, leading to the rather weak fluorescence of ATD-Cl, with an excitation wavelength of 465 nm. Meanwhile, via nucleophilic substitution reaction, the sulfhydryl group originating from GSH can replace the -Cl moiety, initiating the ICT process and the enhancement of fluorescence emission. Through the quantification of the fluorescence change, the concentration of GSH can then be monitored, and the limit of detection can be as low as 89 nM. In addition to the on–off mode, GSH sensing based on the off–on fluorescence change was also presented via the nucleophilic addition reaction (Figure 3B). In this case, a pyronine-based fluorescent compound with the addition of a Ge atom was reported (GeP) [66]. With the introduction of the Ge atom, the charge density of the carbon at the position 9 of pyronine was greatly increased, making it an active site for the occurrence of nucleophilic addition. Therefore, in the presence of GSH, the reaction progressed; meanwhile, the conjugation skeleton of the fluorescent probe was disrupted, resulting in GSH-concentration-dependent fluorescence quenching with an excitation wavelength of 595 nm. The limit of detection for GSH was demonstrated to be 70 nM.

**Figure 4:**
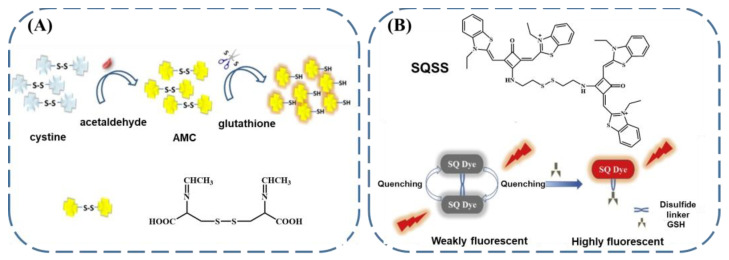
Schematic representation of the fluorescent sensing of GSH using (**A**) AMC (reproduced with permission from [67], Copyright 2018, Elsevier B.V.) and (**B**) SQSS, taking advantage of the reductive nature of GSH (reproduced with permission from [68], Copyright 2019, Elsevier B.V.).

The reductive nature of GSH is another factor that can be employed for the design of fluorescent GSH sensors. Wang et al. prepared an acetaldehyde-modified cysteine (AMC) probe [67]. This probe exhibited fluorescence caused by the n–π* transition of the Schiff base bonds. However, the fluorescence was relatively weak due to the close linking of the Schiff base bonds by disulfide bonds. Meanwhile, in the presence of GSH, the GSH can cleave the disulfide bond of the AMC. Hence, the fluorophores were diffused apart and the fluorescence quenched by aggregation was restored, with an excitation wavelength of 464 nm (Figure 4A). Through the evaluation of the fluorescence changes, GSH could be detected, and the limit of detection was confirmed to be 36 μM. The disulfide–thiol exchange reaction provides an alternative approach for GSH sensing. Xu’s group designed a squaraine-based fluorescence sensor (SQSS) [68]. As shown in Figure 4B, the SQSS featured two squaraines, which were closely linked with disulfide bonds. In the initial state, the fluorescence of the SQSS was almost quenched due to the aggregation-induced quenching and FRET. Since GSH can undergo disulfide–thiol exchange with the SQSS, the squaraines were then distanced from one another in the presence of GSH, leaving the recovery of the fluorescence with an excitation wavelength of 610 nm. In this case, the limit of detection for GSH was verified to be 0.15 μM.

In brief, for sensors based on organic fluorescent compounds, the fluorophores can be flexibly chosen, the output signals are easy to modulate, and the fluorescence changing mode can be designed as desired. In addition, due to the dramatic changes in the fluorescence properties for organic compounds before and after reaction with GSH, the sensors developed with this methodology are usually endowed with high sensitivity (less than 1 μM), which makes them promising in practical biological applications. Moreover, relatively high selectivity is often achieved thanks to the requirement of specific reaction activity for interfering agents. However, high concentrations of other biothiols—for example, cysteine (Cys) and homocysteine (Hcy)—may affect the accuracy of the sensing results. Despite having several advantages, the tedious process of organic synthesis may be one factor that limits the further boosting of organic fluorescent GSH sensors. In addition, regarding biological applications, the lack of targeting capability of the small-molecule fluorescent compounds towards the investigated tissue(s) remains a problem as well.

### 3.2. Fluorescent GSH Sensing via Its Interaction with Nanomaterials

A number of nanomaterials have been demonstrated to feature fluorescence, such as quantum dots and gold clusters [69,70]. It has been demonstrated that the fluorescence intensity of the nanomaterials is closely related to their structures. Therefore, through the interruption of the inner or functional surface structure by GSH, the nanomaterials can be used as fluorescent sensors for GSH [71,72].

**Figure 5:**
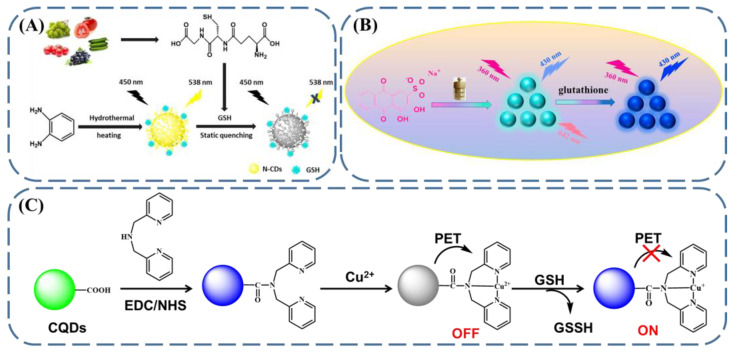
Schematic illustration of carbon-dot-based fluorescent GSH sensing through (**A**) surface attachment of GSH (reproduced with permission from [73], Copyright 2020, Elsevier Ltd.), (**B**) disulfide cleavage by GSH (reproduced with permission from [74], Copyright 2020, American Chemical Society), and (**C**) metal ion reduction by GSH (reproduced with permission from [75], Copyright 2016, Elsevier B.V.).

Carbon dots, as one of the well-known nanomaterials with tunable fluorescence, have received plenty of attention recently. The fluorescent performance of carbon dots has been demonstrated to be supported by the conjugated π-domains of the carbon core, the surface functional groups, the involvement of organic fluorophores, the doping element, etc. Therefore, through the manipulation of the above factors by GSH, the fluorescence of carbon dots can be regulated and, in turn, the carbon dots can be simply employed as fluorescent GSH sensors. Yang et al. designed nitrogen-doped carbon dots (N-CDs) with phenylenediamine as a precursor (Figure 5A) [73]. The N-CDs were demonstrated to have a yellow emission, at 538 nm, with an excitation wavelength of 450 nm. However, in the presence of GSH, the GSH molecules were attached to the surface of the carbon dots, which induced changes in the original surface properties of the N-CDs and resulted in the fluorescence quenching via a static mechanism. In this way, the N-CDs showed high promise for the detection of GSH in vegetables and fruits, with a low detection limit (0.059 μM) and high selectivity. Another example was presented by Shuang et al. (Figure 5B) [74]. In this case, carbon dots with dual emission, at 430 nm and 642 nm, and with an excitation wavelength of 465 nm, were prepared with alizarin carmine. Due to its reductive nature, GSH can reduce the disulfide bonds located on the surface of carbon dots, thereby inducing the disruption of the surface structure. Furthermore, it was demonstrated that, by taking advantage of the NH group, the GSH can interact with the oxygen-containing groups on carbon dots and cause their aggregation. Supported by the above two factors, the emission at 430 nm was increased while that at 642 nm was decreased in the presence of GSH, providing an intrinsic fluorescent GSH sensor in a ratiometric manner. In addition, carbon quantum dots functionalized with the ligands of bis(3-pyridylmethyl)amine (BPMA-CQDs) were fabricated (Figure 5C) [75]. With the coordination of Cu(II), the fluorescence of BPMA-CQDs/Cu(II) was quenched due to PET. Thanks to its reductive ability, the GSH can reduce Cu(II) to Cu(I), preventing the PET process and inducing the fluorescence enhancement. With this sensor, the GSH can be discriminated with a low detection limit (0.31 μM) and high selectivity among amino acids and metal ions.

The nanomaterial-based fluorescent GSH sensors have endowed the area of GSH sensing with several advantages. First of all, the fluorescence of nanomaterials is sensitive to their structure, their surface functionality, and even their aggregation state. In addition to covalent bonding interactions, non-covalent bonding interactions between nanomaterial-based sensors and GSH could induce fluorescence changes as well, endowing the corresponding sensors with relatively high sensitivity and selectivity. Secondly, nanomaterials exhibit passive targeting capability for tumor sites, enabling their good bioavailability and potential for high-resolution imaging and diagnosis of cancer in vivo. However, for biological applications, the degradability issue of inorganic nanomaterials should be paid attention to.

### 3.3. Fluorescent GSH Sensing via Indicator Displacement Assay

**Figure 6:**
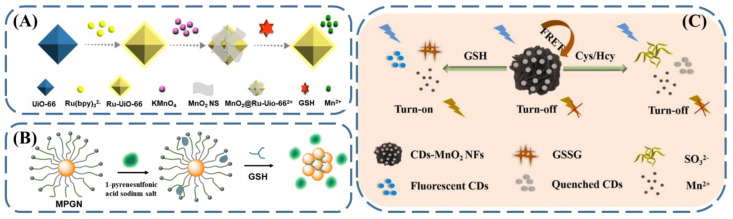
Schematic illustration of fluorescent GSH sensing using indicator displacement assay with (**A**) metal–organic frameworks (reproduced with permission from [78], Copyright 2019, American Chemical Society.), (**B**) monolayer-protected gold nanoparticles (reproduced with permission from [79], Copyright 2022, Elsevier B.V.), and (**C**) MnO_2_-based nanocomposites (reproduced with permission from [80], Copyright 2022, Elsevier B.V.).

Indicator displacement assay is another common strategy for fluorescent GSH sensing [76,77]. Using this methodology, the interaction between GSH and specific species—usually nanomaterials—can be indicated by versatile fluorescent indicators. Taking advantage of the fluorescence changes, the amount of GSH can be quantified. Wang et al. presented this idea by combining luminescent metal−organic frameworks (Figure 6A), which were prepared with Ru-(bpy)_3_^2+^-coated UiO-66 and manganese dioxide (MnO_2_) [78]. Due to the wide UV–Vis absorption of MnO_2_, after coating on the surface of Ru-(bpy)_3_^2+^-UiO-66, the fluorescence of Ru-(bpy)_3_^2+^-UiO-66 was significantly quenched, resulting in a low background platform. However, when mixed with GSH, thanks to its reductive nature, GSH could efficiently reduce MnO_2_ to Mn^2+^, which enabled the recovery of the fluorescence of Ru-(bpy)_3_^2+^-UiO-66, with an excitation wavelength of 420 nm. In this way, the concentration of GSH presented could be quantified from the increased fluorescence intensity, and the limit of detection was demonstrated to be 0.28 μM. Further works expanded this principle with various kinds of nanomaterials, such as metal nanoclusters and graphene. Among these, monolayer-protected gold nanoparticles (MPGNs) have received enormous attention due to their good biocompatibility, flexible functionalization, and ease of preparation. Our group fabricated MPGNs coated with trimethylammonium-based cationic ligands (Figure 6B) [79]. In view of the high affinity between the cationic MPGNs and anionic 1-pyrenesulfonic acid sodium salt, the fluorescence resonance energy transfer occurred, and the fluorescence of the 1-pyrenesulfonic acid sodium salt bound on the surface of MPGN was quenched. When the MPGN-1-pyrenesulfonic acid sodium salt nanoplatform with a low fluorescence background was mixed with GSH, the GSH bound to the surface of the gold nanoparticles via crosslinking and initiated the release of the 1-pyrenesulfonic acid sodium salt to a free molecule state, simultaneously causing the recovery of fluorescence, with an excitation wavelength of 350 nm. With this simple design, GSH was detected with high sensitivity (limit of detection: 1.1 μM) and excellent selectivity among amino acids.

For the detection of GSH, especially in biological systems, cysteine (Cys) and homocysteine (Hcy) usually provide interference due to their similar functionality; therefore, the development of sensing approaches that can discriminate GSH in the presence of Cys/Hcy is of significant importance. With this idea in mind, manganese dioxide nanoflowers (MnO_2_ NFs) were coated with carbon dots through an amidation coupling reaction (Figure 6C) [80]. Similar to the above example, the fluorescence of the carbon dots was quenched by MnO_2_ NFs via FRET. When this platform was mixed with GSH, the GSH reduced the MnO_2_ to Mn^2+^, again leading to an increase in the fluorescence intensity at an excitation wavelength of 380 nm. Meanwhile, in the case of Cys or Hcy, although both of them can induce the decomposition of MnO_2_ NFs, instead of the disulfide bond, their reduction products were demonstrated to be SO3^2−^, which can quench the fluorescence of carbon dots to a larger degree, further decreasing the intensity of the fluorescence. Taken together, the two opposite fluorescence response modes observed for MnO_2_ NFs make them promising candidates for the fluorescent sensing of GSH in practical applications.

In the indicator displacement assay strategy, a higher degree of flexibility is offered for fluorescent GSH sensing. To start with, diverse indicators with high fluorescent quantum yields and emission colors can be flexibly chosen to improve the sensing sensitivity. In addition, when multiple indicators with diversified fluorescence channels are employed simultaneously, fluorescent sensor arrays endowed with high selectivity for GSH can be developed. Moreover, as the displacement event is usually actuated by non-covalent interactions, reversible fluorescence changes can be achieved in the presence and absence of GSH. Therefore, undoubtedly, this approach provides the possibility for in situ monitoring of GSH in vivo. However, the stability of fluorescent nanomaterial–indicator sensing platforms during blood circulation and their related bioavailability should be considered for biological applications.

Above all, taking advantage of fluorescent output signals originating from organic compounds, fluorescent nanomaterials, or fluorescent indicators, GSH can be sensitively and selectively detected. In addition to the abovementioned examples, the limit of detection and selectivity of other GSH sensors are listed in Table 1 to further prove their potential in practical applications.

## 4. Fluorescent GSH-Sensing-Actuated Bio-Applications

Taking into account that GSH plays a crucial role in biological processes and, more interestingly, that there is a significant discrepancy in GSH concentration between normal cells and certain diseased cells, intracellular GSH sensing is becoming a hot topic. Currently, enzymatic assays (e.g., Ellman’s assay) for GSH detection are commercially available. Although these methods are endowed with satisfying sensitivity, most of them can only be used in vitro, as the output information that they provide is the intensity of UV–Vis signals. Therefore, fluorescent GSH sensing, which offers the possibility of spatial and temporal resolution, can greatly benefit GSH imaging, cancer cell recognition, and even the monitoring of stroke or neurological diseases [91]. In this section, the possible bio-applications prompted by GSH sensing are discussed.

### 4.1. GSH Imaging

Fluorescence imaging is an essential way to study the GSH-related physiological processes. To apply GSH imaging in cellular, living systems—or even subcellular systems—with fluorescent GSH sensors, several factors need to be kept in mind: (1) the degree of fluorescence enhancement or the sensitivity of the probe to GSH, (2) the background of the GSH-sensing probe, (3) the quantitative evaluation, (4) the influence of possible interference, (5) the biocompatibility, (6) the bioavailability, and (7) the biological stability of the probe [92,93,94].

**Figure 7:**
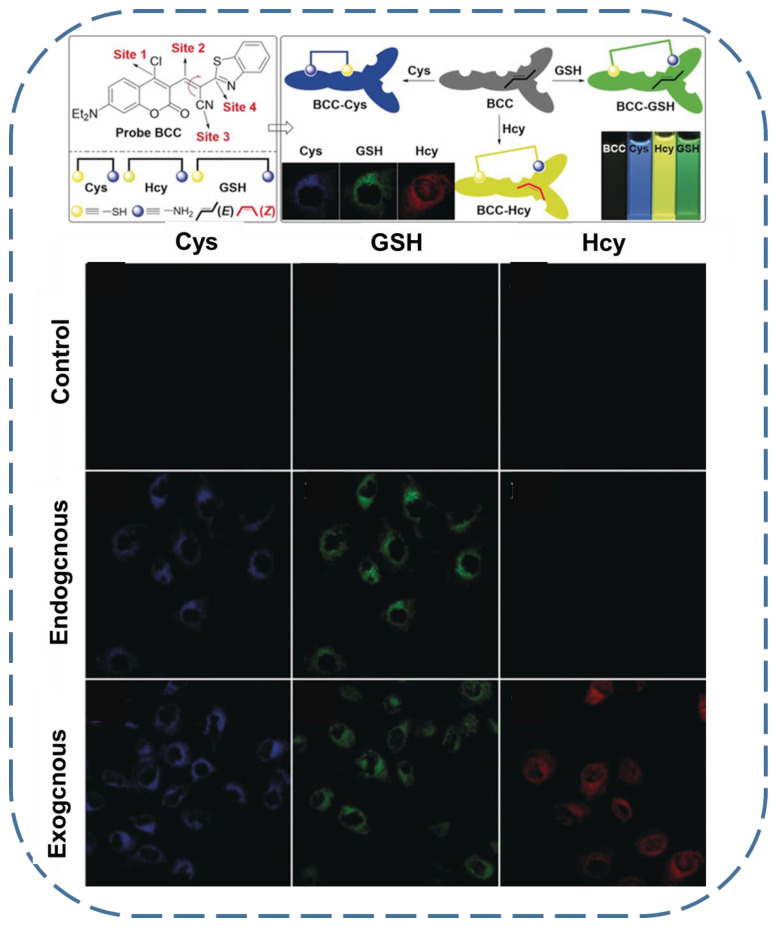
Upper: the molecular structure of Probe BCC and the mechanism for multiple binding sites inducing selective recognition of GSH. Lower: the differentiated fluorescence emission of Probe BCC in the presence of Cys, GSH, and Hcy in BEL-7402 cells (reproduced with permission from [96], Copyright 2018, Wiley-VCH Verlag GmbH & Co. KGaA).

As addressed above, the selective imaging of GSH, with negligible interference from other biothiols, is an important factor that needs to be taken into account for the practical application of fluorescent GSH sensors. In biological systems, Cys and Hcy are two common kinds of thiols that may affect the accuracy of GSH imaging results [95]. Thanks to the slight differences in their molecular structures, a multisite binding fluorescent probe (Probe BCC) was developed by Yin’s group (Figure 7) [96]. The core fluorophore of the probe is coumarin. While in different positions, the coumarin is modified with n-butylthio groups, α,β-unsaturated C=C bonds, and cyano groups as binding sites for nucleophilic substitution, Michael addition or amino addition, and amino addition, respectively. Therefore, the reaction of the probe with GSH, Cys, and Hcy was imparted with different mechanisms and, of course, resulted in products with different conjugation sections, i.e., fluorescence properties. The biological results further confirmed that the abovementioned fluorescent probe can differentiate the GSH from Cys and Hcy in BEL-7402 cells via the monitoring of the different fluorescence channels.

**Figure 8:**
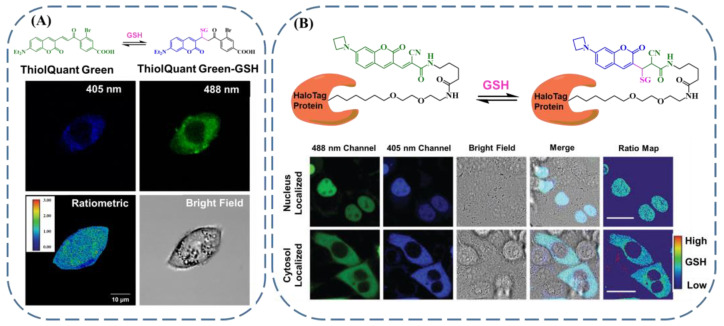
(**A**) ThiolQuant-Green-based reversible reaction with GSH and related ratiometric quantification of GSH in living cells (reproduced with permission from [97], Copyright 2014, American Chemical Society.). (**B**) GSH quantification with subcellular resolution based on HaloTag technology combined with a reversible reaction (reproduced with permission from [98], Copyright 2019, Mary Ann Liebert, Inc.).

Quantitative imaging of GSH in live cells can significantly benefit the deep understanding of a series of pathological processes. Wang et al. creatively developed a reversible-reaction-based GSH sensor taking advantage of ThiolQuant Green (Figure 8A). [97] The ThiolQuant Green featured green emission (590 nm) with an excitation wavelength of 479 nm. In the presence of GSH, the ThiolQuant Green, which served as a Michael acceptor, underwent Michael addition with GSH, resulting in the product of ThiolQuant Green–GSH, which was imparted with blue emission (463 nm) with an excitation wavelength of 406 nm. Through the regulation of the equilibrium constant, this sensor can be employed for the quantitative discrimination of concentrated GSH in living cells in a ratiometric manner while using a small amount of ThiolQuant Green. The quantitative GSH sensing with spatial and temporal resolution provides the most direct evidence for the understanding of the biological functions of GSH. Jiang et al. reported a novel strategy in this respect [98]. They elaborately combined the HaloTag protein and a reversible Michael-addition-reaction-based fluorescent GSH sensor (Figure 8B). Similar to the above example, the GSH-related reversible reaction offered ratiometric output signals, which could be used for the quantitative discrimination of GSH inside cells. When different HaloTag proteins were employed for the targeting of various organelles, by comparing the ratio of the fluorescence signal between the probe and the product, the concentration gradient of GSH located in the nucleus and cytosol was demonstrated to be inappreciable. Moreover, upon treatment with hydrogen peroxide, buthionine sulfoximine, tunicamycin, and nelfinavir, the GSH concentrations in the nucleus and cytosol experienced similar changes for HeLa cells. The subcellular imaging of GSH with spatial and temporal resolution provided by this approach will undoubtedly spur progress GSH-sensing-related biological applications. Taken together, to achieve quantitative GSH imaging with spatial and temporal resolution, the following two factors should be considered: (1) the precise quantification parameter in a suitable concentration range, and (2) the targeting capability for the investigated cells or organelles and their resultant bioavailability.

### 4.2. Cancer Cell Recognition

**Figure 9:**
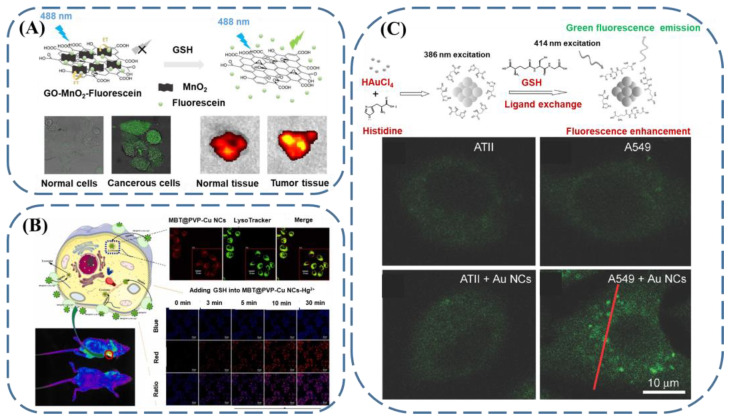
Fluorescent GSH-sensing-promoted cancer cell recognition with (**A**) MnO_2_-based nanocomposites (reproduced with permission from [99], Copyright 2018, Elsevier Inc.), (**B**) Cu nanoclusters (reproduced with permission from [100], Copyright 2022, Elsevier B.V.), and (**C**) Au nanoclusters (reproduced with permission from [101], Copyright 2014, Wiley-VCH Verlag GmbH & Co. KGaA.).

Taking advantage of the concentration discrepancy of GSH between tumor tissue and healthy tissue, cancer cell recognition can be achieved with fluorescent GSH sensors. Wu et al. developed an elaborate method for cancer cell recognition by monitoring the GSH contents [99]. They prepared nanocomposites by encapsulating fluorescein on graphene oxide–MnO_2_ (Figure 9A). Through the FRET, the fluorescence of fluorescein was quenched by MnO_2_ from graphene oxide–MnO_2_. Meanwhile, in the presence of GSH, the MnO_2_ decomposed and contributed to the recovery of the fluorescence. Encouraged by the fact that the nanocomposites exhibited excellent cytocompatibility, the graphene oxide–MnO_2_–fluorescein was testified in living systems and provided the possibility of intracellular imaging. Even more fascinating, when the nanocomposites were incubated in cancerous and healthy mice, due to the higher amounts of GSH in tumor tissue, twofold brighter fluorescence was observed in the tumor tissue than in the normal tissue, offering the promise of GSH-sensing-actuated cancer diagnosis. Zhang et al. [100] reported a fluorescent nanocluster, MBT@PVP-CuNCs (Figure 9B), which was composed of polyvinylpyrrolidone (PVP), 2-mercaptobenzothiazole (MBT), and copper nanoclusters (CuNCs). It was found that the Hg^2+^ could effectively diminish the fluorescence ratio at F_585_/F_432_, in comparison with a series of metal ions, amino acids, and saccharides. Furthermore, taking advantage of the strong affinity between GSH and Hg^2+^, the Hg^2+^ was displaced from the MBT@PVP-CuNCs by GSH and the fluorescence of the MBT@PVP-CuNCs was recovered, making MBT@PVP-CuNCs-Hg^2+^ an appealing candidate for GSH detection. More importantly, the tumor cell recognition capability of MBT@PVP-CuNCs was demonstrated both in vitro and in vivo.

Gold nanoclusters (Au NCs) are known for their excellent biocompatibility and as attractive candidates for bio-applications. With the mixing of HAuCl_4_ and histidine, Au NCs with a size of ~3 nm were prepared (Figure 9C). Chen et al. found that the as-obtained Au NCs had relatively weak fluorescence [101]. Meanwhile, in the presence of GSH, the Au-S bonds were formed and charge transfer from the ligands to the metal cluster occurred, resulting in the marked enhancement of fluorescence. As the common biothiols Cys and Hcy did not show any obvious effect on the fluorescence change, the as-reported Au NCs were tested in biological systems, and a selective fluorescence increase in tumor cells was achieved, providing an alternative approach for cancer diagnosis.

### 4.3. Other Bio-Applications

**Figure 10:**
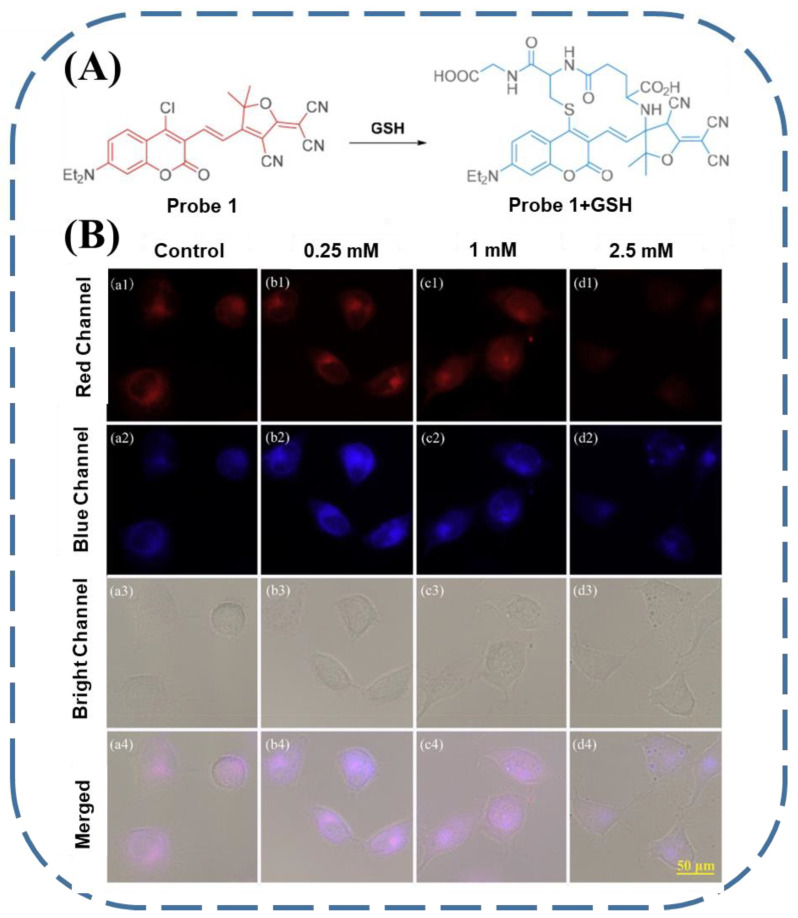
(**A**) The molecular structure of Probe 1 and the related mechanism for the detection of GSH. (**B**) The discrimination of various amounts of ROS in BEL-7402 cells by Probe 1 (reproduced with permission from [102], Copyright 2020, Elsevier B.V.).

The monitoring of ROS-induced redox imbalance has been achieved with fluorescent GSH sensors. In this respect, Yin’s group synthesized a coumarin probe functionalized with chloride and 2-dicyanmethylene-3-cyano-4,5,5trimethyl-2,5-dihydrofuran (Probe 1) [102]. This probe could selectively detect GSH from other biothiols according to the substitution–cyclization cascade reaction (Figure 10A). When applied in a cellular system, the fluorescence of the above probe was attenuated along with the increased ROS treatment (Figure 10B), making it a proper candidate for the recognition of excessive ROS in vivo.

**Figure 11:**
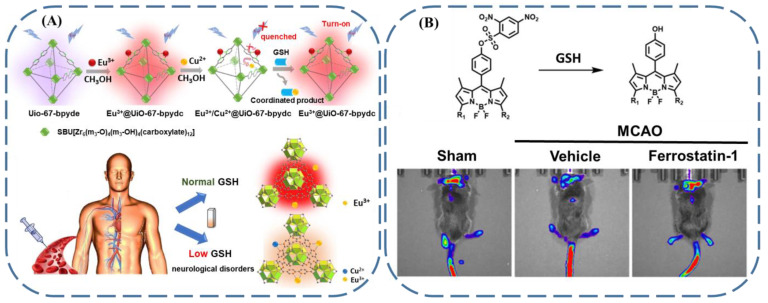
(**A**) Schematic illustration of a UiO-67-bpyde-based fluorescent GSH sensor and related principle for the diagnosis of neurological diseases (reproduced with permission from [103], Copyright 2019, Elsevier Inc.). (**B**) Upper: the molecular structure of a BODIPY-based probe and its mechanism for the sensing of GSH. Lower: the in vivo monitoring of stroke and ferroptosis with an MCAO model (reproduced with permission from [104], Copyright 2022, Elsevier B.V.).

Reduced amounts of GSH in serum are related to a series of neurological diseases. According to this fact, Qian’s group designed a probe of Eu^3+^/Cu^2+^@UiO-67-bpydc (Figure 11A), of which the fluorescence of the Eu^3+^ was quenched by Cu^2+^ [103]. Since GSH has the ability to coordinate with Cu^2+^, it could remove Cu^2+^ from the Eu^3+^/Cu^2+^@ UiO-67-bpydc probe and induce the fluorescence enhancement. When this GSH sensor was applied in fetal bovine serum, the concentration of GSH in the serum could be accurately fitted with the intensity of fluorescence, confirming its suitability for the diagnosis of neurological diseases.

The diagnosis of cerebral ischemia–reperfusion injury has also been accomplished by taking advantage of fluorescent GSH sensors. This breakthrough was reported by Gu et al. [104], who designed an elaborate series of BODIPY-based fluorophores functionalized with a 2,4-dinitrobenzenesulfonate group (Figure 11B). Because of the strong electron-withdrawing capability of the 2,4-dinitrobenzenesulfonate group, the PET process occurred, and the fluorescence of the as-prepared fluorophores was quenched. Due to the ability to cut off the 2,4-dinitrobenzenesulfonate group from the probes, the GSH could be quantified from the fluorescence enhancement. Even more appealing, in a middle cerebral artery occlusion (MCAO) model that can stimulate stroke, the fluorescence signal of the probe changed along with the variation in the GSH concentration, and the detection of stroke was achieved. Moreover, by adding ferrostatin-1 to the MCAO model, the fluorescence enhancement verified that the stroke was accompanied by ferroptosis.

## 5. Conclusions and Outlooks

In view of the recent progress, fluorescent methods have been proven to be an effective approach for the quantitative detection of GSH. Through covalent reactions or non-covalent interactions, the fluorescent sensing platforms for GSH can use fluorescent organic compounds, fluorescent nanomaterials, or even fluorescent indicators coated with non-fluorescent nanomaterials. Generally, due to the high sensitivity of fluorescent methods, the limit of detection for GSH is usually less than 1 μM. In addition, due to the specific functionality of the thiol group, in most cases, the GSH can be selectively discriminated among amino acids and metal ions. As a result of the high sensitivity and selectivity of fluorescent GSH sensors, quantitative imaging with spatial and temporal resolution was achieved. Moreover, methodologies related to fluorescent GSH sensing have also achieved cancer diagnosis and the monitoring of neurological disease and stroke. Though great efforts have been made, space for future improvement still remains. (1) One of the obstacles that limit the practical application of fluorescence imaging is the disturbance of the tissue’s autofluorescence. To minimize the effect of tissue autofluorescence on the GSH imaging, the design of NIR fluorescent imaging systems could be a meaningful direction. (2) As the concentration of GSH is relevant to cancer diagnosis, and the overexpressed GSH can scavenge antitumor species (e.g., reactive oxygen species), the combination of GSH sensing and GSH depletion performance could be a popular topic to facilitate cancer diagnosis and therapy simultaneously. (3) The development of fluorescent GSH sensors with spatial and temporal quantification capability in cellular or even subcellular systems is challenging. Though examples have been given, the in situ monitoring of GSH in specific tissues, which benefits both disease diagnosis and related therapy, will offer new opportunities in this field. (4) In addition to focusing on the functionality of GSH sensors and their related biological applications, the bioavailability of corresponding sensing platforms should be paid attention to, and novel strategies for their improvement should be developed.

## Figures and Tables

**Table 1:** The structure, limit of detection, and sensitivity of representative fluorescent GSH sensors.

Structure	Limit of Detection	Selectivity
CBF_3_ [62]	9.2 nM	Amino acids, anions, and amines
Lyso-RC [61]	27 nM	Cys/Hcy, GSH, and H_2_S
NH_2_-UiO-66@AgNPs [81]	79 nM	Amino acids
BSA@AuNCs–MnO_2_ [82]	1000 nM	Ions and amino acids
UCNPs [83]	200 nM	Ions and amino acids
UCNP@RBD probe [84]	50 nM	Serum samples and urine samples
Cdot-MnO_2_ nanostructures [85]	19,000 nM	Amino acids
GODs-MnO_2_ [86]	48 nM	Ions and amino acids
carbon dots–MnO_2_ [87]	300 nM	Electrolytes, amino acids, and proteins
Murexide-Hg^2+^ system [88]	100 nM	Amino acids and anions
ACD [89]	6 nM	Cys, Hcys, and GSSG
Graphene quantum dot−MnO_2_ [90]	150 nM	Inorganic salts, metal ions, amino acids, and proteins,
